# Mitral valve repair or replacement. How long is this feud to last?

**DOI:** 10.1111/jocs.16479

**Published:** 2022-04-01

**Authors:** Michele Di Mauro, Marco Cargoni, Roberta Liberi, Roberto Lorusso, Antonio M. Calafiore

**Affiliations:** ^1^ Cardio‐Thoracic Surgery Department, Heart & Vascular Centre, Cardiovascular Research Institute Maastricht (CARIM) Maastricht University Medical Centre (MUMC) Maastricht The Netherlands; ^2^ Department of Cardiac Anaesthesia and Intensive Care “Mazzini” Hospital Teramo Italy; ^3^ Department of Heart Disease “SS Annunziata” Hospital Chieti Italy; ^4^ Division of Cardiac Surgery A “Henry Dunant” Hospital Athens Greece

**Keywords:** annuloplasty, ischemic mitral regurgitation, valve repair/replacement

## Abstract

Choosing to perform mitral valve (MV) repair or replacement remains a hot and highly debated topic. The current guidelines seem to be conflicting in this specific field and the evidence at our disposal are scarce, only one small randomized trial and few larger retrospective studies. The meta‐analysis by Gamal and coworkers tries to summarize the current evidence, concluding that MV replacement for the treatment of ischemic mitral regurgitation (MR) is at least as safe as repair and certainly offers a more stable result over time than the latter. Obviously, the implantation of a prosthesis, especially a mechanical one, brings with it a series of problems, such as anticoagulation and, above all, a possible lack of ventricular remodeling, especially if a chordal sparing replacement is not performed. It must be said, on the other hand, that isolated annuloplasty cannot act as a counterpart to replacement, because ischemic MR cannot be considered only an annular disease. Therefore, wanting to mimic the nature that, after an infarction, enacts a series of changes involving also the mitral leaflets and chordae, the surgeons are called to act also on these two entities and not only to downsize the annulus. In a nutshell, a procedure should not be opposed in a fundamentalist way to another one, but we must accept the concept of armamentarium where both procedures are present and tail on the single patient, and also on the surgeon's expertize, the technique guaranteeing the best possible result.

1

Ennio Flaiano, an important Italian writer said “I have few ideas but confused” and we strongly believe that it is really appropriate to make this quote talking about the treatment of ischemic mitral regurgitation (MR). Indeed, comparing the two most important guidelines on the management of heart valve disease, the European^1^ and the North American^2^ ones, it is possible to read two different recommendations. The former states that although the surgical approach has to be tailored to the individual case, in selected patients without advanced left ventricular (LV) remodeling, mitral valve (MV) repair with an undersized complete rigid ring restores valve competence, improves symptoms, and results in reverse LV remodeling. Conversely, even if valve replacement avoids recurrence of MR, this does not translate into better LV reverse^3^ From this, it is possible to infer that they lean toward a conservative procedure over MV replacement.

The AHA/ACC guidelines,^2^ on the other hand, unequivocally recommend mitral replacement with chordal sparing over annuloplasty, with a 2b class of evidence.^3^, ^4^, ^5^

So apparently choosing or not choosing one procedure rather the other has become a geographical issue.

In this confusing scenario, the meta‐analysis of Gamal and coworkers^6^ tries to thin out at least somewhat the fog. Among 2987 records initially identified, the authors report pooled results of 14 studies including only one randomized trial.^7^ Pooled analysis on mortality clearly confirms that MV replacement is as safe as MV repair [OR (95% CI): 0.66 (0.41–1.07)], as already reported in both clinical trial and some large retrospective analyses, already cited by AHA/ACC guidelines.^2^, ^3^, ^4^, ^5^, ^7^

Likewise, MV repair is confirmed to be a harbinger of a higher rate of late MR recurrence (pooled rate: 200 [34.4%] vs. 22 [3.9%]; OR: 16.8 [5.07–55.7], *p* = .00001) although this is not matched by a significant increase in the rate of reinterventions (55 [7.0%] and 40 [6.0%]; OR [95% CI]: 1.34 [0.87–2.06], *p* = .19). This difference can obviously be explained by the fact that MR recurrence includes by definition also moderate grade that is not indicated for surgery in the first instance, let alone as reintervention, and by the fact that patients who have undergone surgery often refuse to undergo a new procedure and choose the path of medical therapy.

Conversely, repair seems to result in improved LV remodeling with consequent improvement in the patient's functional capacity.

However, the latter finding could be related to the fact that not everyone performs mitral replacement with sparing of the subvalvular apparatus as suggested by many surgeons.^8^

Indeed, in CTSNET trial,^7^ where 95.4% of those receiving valve replacement had also a chordal‐sparing procedure, the mean change of LV end systolic volume from baseline was −9.0 ml/m^2^ and −6.5/m^2^), not statistically significant (*p* = .19).

Similarly, in a large multicenter Italian study^5^ in which almost all the patients had MV replacement with preservation of valvular‐subvalvular continuity, LV remodeling in terms of ejection fraction was similar in both groups (*p* = .66).

From what has just been said, it would seem that the best choice is to replace the MV, in the presence of ischemic etiology, because at equal survival, repair results in a higher recurrence rate. Yet, as we have seen, the European guidelines continue to support the choice of repair as a priority without consistent scientific evidence (rather, the opposite).

To best address the issue of high MR recurrence rate, we must return for a moment to the definition of secondary (or ischemic, predominantly) mitral insufficiency and change the paradigm of surgical treatment of this disease. In a recent editorial,^9^ we have already pointed out that ischemic MR represents a unique situation in cardiac surgery. In fact, although MR has long been considered a ventricular problem, suggesting that the valve leaflets and chordal structures are merely innocent bystanders, this concept needs to be challenged.

In ischemic MR, a set of adaptive processes termed “mitral plasticity” takes the field aimed to reduce the amount of regurgitation. Unfortunately, the mitral plasticity is not always able to achieve the goal of balancing higher tenting forces and papillary muscles displacement.

Mimicking what the nature does, surgery may adopt some surgical steps to go along with the annuloplasty, such as anterior leaflet augmentation and second order chordal cutting.^10^

We believe it is time to end this feud between repair and replacement (Figure 1). It is now obvious that they are both worthwhile surgical approaches, and this is also confirmed by the meta‐analysis by Gamal et al.^6^

**Figure 1:**
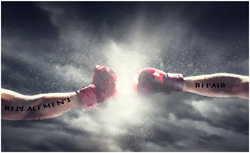
The feud between mitral valve repair and replacement

Already at the beginning of the 2000s, we proposed a simple echocardiographic parameter, the coaptation depth, to choose between the two techniques, reserving replacement for patients with worse LV remodeling, going against the dogma according to which MV repair should be the predominant choice to be adopted.^11^ Today we know that isolated annuloplasty may not be enough to reach a long‐term stability and competence of the valve and, in these cases, we can choose between a more complex MV repair procedure that includes also a valvular and subvalvular procedure, or valve replacement. In a nutshell, as often happens in surgery, a procedure should not be opposed in a fundamentalist way to another one, but we must accept the concept of armamentarium where both procedures are present and tail on the single patient, and also on the surgeon's expertize, the technique guaranteeing the best possible result. But the struggle to generate a single, long‐lasting, and effective technique in ischemic MR will certainly continue…. at least, in the guidelines recommendations.
